# Mangrove Habitats in Zhanjiang of South China: A Potentially High‐Risk Environment for Breeding Birds

**DOI:** 10.1002/ece3.72651

**Published:** 2025-12-08

**Authors:** Yiming Liu, Jianping Liu, Wei Liang

**Affiliations:** ^1^ Zhanjiang Mangrove National Nature Reserve Zhanjiang Guangdong China; ^2^ College of Biological Sciences and Engineering North Minzu University Yinchuan China; ^3^ Ministry of Education Key Laboratory for Ecology of Tropical Islands, Key Laboratory of Tropical Animal and Plant Ecology of Hainan Province College of Life Sciences, Hainan Normal University Haikou China

**Keywords:** breeding bird, crab predation, mangrove, nest predation

## Abstract

Nest predation is one of the most important causes of breeding failure in birds, and the risk of predation is closely related to nest site selection. To maximize site suitability, birds should choose safe locations for nest site to reduce the risk of nest predation. Mangroves and mudflats are very important feeding and wintering grounds for many birds; however, we still know little about the risk of nest predation experienced by birds breeding in mangrove habitats. From August to December 2019, we studied nest predation rates and predator types in mangroves by setting up artificial nest experiments in the Zhanjiang Mangrove National Nature Reserve in Guangdong Province, south China. The results showed that the nest predation rates in the Jiulongshan mangrove area in the reserve were substantially higher than those in the Caoyang mangrove area during the first 3 days for both the artificial nests on trees and on the ground. However, the nest predation rates were 100% for the experimental nests in both mangroves within 7 days. There were diverse predators, including the greater coucal ( *Centropus sinensis* ), Oriental magpie‐robin ( *Copsychus saularis* ), white‐breasted waterhen ( *Amaurornis phoenicurus* ), mangrove crabs (*Chiromantes dehaani*), and rats. We recorded a total of 44 nests. The predation rate of the greater coucal on the experimental nests was as high as 70.45%, while the predation rates of the mangrove crabs and rats on the experimental nests were both 20.45%. Our research indicates that birds breeding in mangrove habitats of the Zhanjiang Mangrove in south China face a relatively high risk of nest predation.

## Graphical abstract

Mangroves are important feeding and wintering grounds for many birds. However, we still know little about the risk of nest predation experienced by birds breeding in mangrove habitats. The nest predation rates were 100% for the experimental nests in mangroves within 7 days, with diverse predators. Birds breeding in Zhangjiang mangrove habitats of south China face a relatively high risk of nest predation.

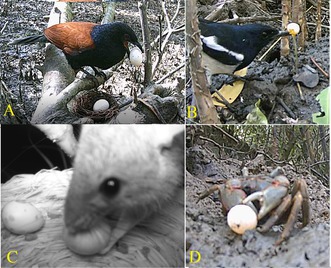

## 1 Introduction

Predation is one of the main causes of nesting failure (Ricklefs 1969; T. E. Martin 1993) and is an important factor affecting the nesting ecology of birds (T. E. Martin 1993, 1995; Parejo and Avilés 2011). Natural selection has led to the evolution of appropriate breeding strategies in birds to reduce the risk of nest predation and maximize their fitness (T. E. Martin 1998; Lima 2009; Hua et al. 2014; Maisey et al. 2016; Zhang et al. 2020). Several studies have shown that birds reduce the risk of nest predation via certain behaviors before nesting, during egg incubation, or during brood rearing (Fontaine et al. 2007; Brawn et al. 2011; Ocampo and Londoño 2015). For example, many birds will prefer to breed in places that are inaccessible to predators or breed at safe and secluded nest sites to reduce the risk of predation (Eggers et al. 2006; Peluc et al. 2008; Wright et al. 2013; Cancellieri and Murphy 2014; Ocampo and Londoño 2015; Macdonald et al. 2016; Jiang et al. 2017; Liu et al. 2025), and some birds even actively guard their nests and engage in aggressive behaviors to deter potential predators (Kostoglou et al. 2020), although there are also many species that build their nests in open and accessible areas, including urban areas (James Reynolds et al. 2019). Overall, each species may select nesting sites based on certain features such as concealment, proximity to food sources, protection from weather, etc. However, there remains limited understanding regarding whether habitats that appear suitable for avian breeding can effectively reduce the risk of nest predation and positively influence reproductive success.

Mangroves are woody plant communities in the intertidal zone of subtropical and tropical coastal areas. As one of the important types of wetlands, they play a key role in maintaining the ecological balance of the coast, reducing the impact of wind and disasters, protecting embankments and shores, and purifying environmental pollution (Furness et al. 1993; Akram et al. 2023). At high tide, mangroves are often submerged by seawater, or only with the canopy exposed; at low tide, the horizontal and vertical growth of mangroves is evident in the mudflats. The abundance of benthic animals such as shrimps and crabs in mangroves provides an abundant food source for birds, making mangroves ideal for foraging, breeding, and wintering grounds for many birds (Way and Heong 1994; Lin 1997; Zou et al. 2001, 2008; Wang and Wang 2007; Zhang et al. 2008; Cheng et al. 2019; Mancini et al. 2023).

In China, there are 445 species of mangrove birds, among which 173 are water birds, accounting for 38.9% of the total number of mangrove birds (Zou 2010). Some surveys have shown that mangrove summer birds are mostly herons, such as the Chinese Pond Heron ( *Ardeola bacchus* ), the Little Egret ( *Egretta garzetta* ), the Green‐backed Heron ( *Butorides striata* ), and the Black‐crowned Night Heron ( *Nycticorax nycticorax* ) (Zou et al. 2001, 2008; Tan et al. 2013; Cheng et al. 2019). Other birds, like the Scarlet Ibis ( *Eudocimus ruber* ), are also common in Brazilian mangroves (Mancini et al. 2023). Larger waterbirds that breed in mangroves, such as herons, are primarily social breeders. As such, their large colonies and sizable nests render them easily detectable by humans (Bellinato and Bogliani 1995). In China, although there are many studies on the community structure and diversity of birds in mangroves, there have been few reports on birds breeding in mangroves (Zhu and Zou 2001; Wong et al. 2004; Liang et al. 2006). Furthermore, there remains a lack of relevant reports identifying the primary predators of mangrove‐breeding birds and quantifying the associated predation risk in China.

While previous studies have documented crabs preying on bird eggs or chicks (Yang et al. 2014; Kwon et al. 2018), and the Zhanjiang mangrove reserve we studied harbors a diverse assemblage of crab species, it remains unknown whether crabs prey on eggs and nestlings of birds breeding in these mangroves and, if so, which crab species are responsible for that. Therefore, an artificial nest predation experiment was set up from August to December 2019 in Zhanjiang Mangrove National Nature Reserve, Guangdong, China. Nest predation rates and predator types in mangroves were studied.

## 2 Materials and Methods

### 2.1 Study Area

The Zhanjiang Mangrove National Nature Reserve is located at the southernmost tip of mainland China (20°15′–21°55′ N, 109°40′–110°55′ E). Patches of mangroves are scattered along the coastal mudflats of Leizhou Peninsula in southwest Guangdong Province that face the South China Sea in the east, with the Beibu Gulf in the west connected to Guangxi and with the Qiongzhou Strait in the south facing Hainan Island across the sea, covering an area of approximately 200 km^2^. The reserve is located in the transition zone from the northern tropics to the southern subtropics and is influenced by the monsoon and marine climates. The average annual temperature is 23°C, with a maximum temperature of 38.8°C and a minimum temperature of −1.4°C. The average annual precipitation is 1534.6 mm, with rainfall concentrated between April and September, which is also the typhoon season (Zou et al. 2008). The coastal tidal patterns are varied, with an average tidal height of approximately 2 m.

### 2.2 Artificial Nest Predation Experiments

**FIGURE 1:**
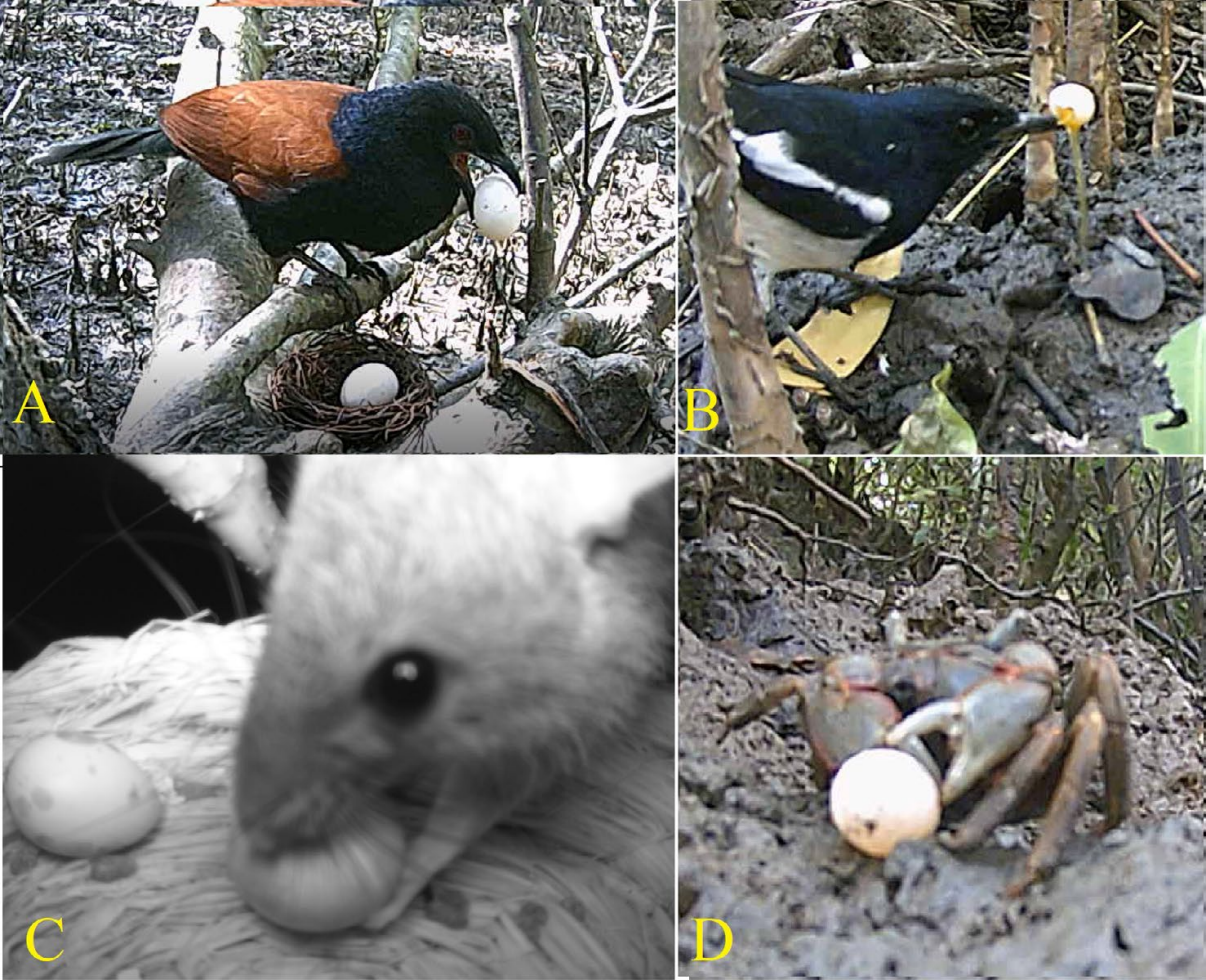
Four main predators in mangroves identified in this study. (A) Greater coucal preying on a pigeon egg, (B) An oriental magpie‐robin preying on a munia egg, (C) A rat preying on a quail egg, and (D) a crab preying on a munia egg.

From August to December 2019, artificial nest experiments were set up in the Jiulongshan and the Caoyang mangrove areas in the Zhanjiang Mangrove National Nature Reserve to study the nest predation rates and predator types in the mangrove forests of the two sites. Artificial nests were woven from rattan (Figure 1A). We placed artificial nests either in trees (*n* = 46 in Jiulongshan; *n* = 20 in Caoyang) or on the ground (*n* = 34 in Jiulongshan; *n* = 20 in Caoyang). Given the average height of the local mangrove species in both areas is 2.5 m, tree nests were positioned at approximately 2 m height with minimal overhead vegetation cover. The reason for choosing this specific height for the nest is that there are more horizontal branches at this level, which makes it convenient to fix the artificial nest. Ground nests also had 0% vegetation coverage within 0.5 m above them. The locations of the artificial nests were randomly chosen, and the minimum distance between nests was 30 m. Placement of experimental nests was based primarily on practicality. For tree nests, we ensured sufficient distance between nests and selected trees that facilitated secure attachment. For ground nests, we chose locations with minimal vegetation cover. The only difference between the artificial nest experiments at the two sites was that quail eggs were placed in the artificial nests in the Jiulongshan mangrove area, whereas pigeon eggs were placed in the artificial nests in the Caoyang mangrove area. Quail and pigeon eggs were both commercially available, with unfertilized eggs being purchased online. The reason for choosing quail and pigeon eggs was primarily because they are similar to the eggs of shorebirds and herons that might breed in mangroves.

In addition, the predation of bird nests by crabs in the Caoyang mangrove area was also investigated, that is, two munia (*Lonchura* spp.) eggs, or a munia egg and a pigeon egg, were placed 10 cm from a crab hole and the predation of bird eggs by crabs was observed and recorded (*n* = 66).

We observed all experimental nests or experimental eggs continuously for 7 days and recorded the outcome of the experimental eggs. In addition, some nests were videotaped to record the predator type (*n* = 44). Pearson's Chi‐squared test was used to compare the predation rates in different experimental groups. All tests were two‐tailed, with a significance level of *p* < 0.05. IBM SPSS 22.0 software (IBM Corp., Armonk, NY, USA) was used for all data analyses.

## 3 Results

For the first 3 days, the predation rates of 65.2% (*n* = 46) and 82.4% (*n* = 34) for the tree and ground nests, respectively, in the Jiulongshan mangrove area were significantly higher than the predation rates of 35% (*n* = 20) and 45% (*n* = 20) for those in the Caoyang mangrove area, respectively (Chi‐square test, *ꭓ* ^2^ = 5.167, df = 1, *p* = 0.032; *ꭓ* ^2^ = 8.145, df = 1, *p* = 0.007). However, the predation rate was 100% for both tree and ground nests at both sites within 7 days.

In the Caoyang mangrove area, the predation rate of the eggs was as high as 77.7% (*n* = 36) within a day of placing two munia eggs near the crab hole, and 100% (*n* = 30) within a day of placing one munia egg and one pigeon egg near the crab hole, and 86.6% (*n* = 30) within a day of placing one pigeon egg.

In total, 44 experimental nests were videotaped, including 14 tree nests, six ground nests, and 24 nests at crab holes (Table 1). Five types of predators were identified, including the greater coucal ( *Centropus sinensis* ) (Figure 1A), Oriental magpie‐robin ( *Copsychus saularis* ) (Figure 1B), mangrove crab (*Chiromantes dehaani*) (Figure 1C), rats (Figure 1D), and white‐breasted waterhen ( *Amaurornis phoenicurus* ). The greater coucal was involved in 70.4% (*n* = 44) of the total occurrences of predation; rats and crabs each accounted for 20.4% (*n* = 44) and 2.2% (*n* = 44), while only one case each was caused by the Oriental magpie‐robin and white‐breasted waterhen, respectively.

**TABLE 1:** Predation of artificial nests by five predators at two mangrove study areas.

Site	Nest location	Experiment	Predator type			Number of videos of predated nests
			Greater coucal	Rat	Crab	
Jiulongshan	On the tree	Two quail eggs	1	2		3
	On the ground			2		2
Caoyang	On the tree	Two pigeon eggs	10	2		11
	On the ground		4			4
	On the crab hole entrance	Two munia eggs	9	1	8	17
		One munia and one pigeon egg	7	2	1	7

*Note:* One of the nests on the ground in Jiulongshan was jointly predated by a rat and a white‐breasted waterhen; one of the nests on a tree in Caoyang was jointly predated by a rat and a greater coucal. In the experiment with two munia eggs near a crab hole, one nest was jointly predated by an Oriental magpie‐robin and a greater coucal; in the experiment with one munia egg and one pigeon egg, one nest was jointly predated by a greater coucal and a rat, and the other was jointly predated by a crab and a greater coucal.

## 4 Discussion

Our study shows that birds breeding in Zhanjiang mangroves face multiple predators. Among them, the greater coucals and rats prey on both ground nests and tree nests. Crabs in mangroves also prey on bird eggs, with the primary predators being Sesarmidae crabs. Nests near crab holes were predated more rapidly than nests at other sites. Our research indicates that birds breeding in Zhanjiang mangrove habitats are exposed to a high risk of predation.

Nest predation is the main cause of life history evolution in birds (Roff 1992). Birds are limited to the vicinity of their nests during the breeding season. In addition, predators can locate breeding parents through their nests. Therefore, breeding birds are highly sensitive to the risk of predation (Lima and Dill 1990). Studies have shown that birds perceive predation risk in their environment and choose to build concealed nests or avoid breeding in high‐risk areas to reduce predation risk when nesting (Eggers et al. 2006; Mönkkönen et al. 2009; Parejo and Avilés 2011; Liu et al. 2025). For example, the dusky warbler ( *Phylloscopus fuscatus* ) secures its nest by building it at increased heights from the ground and in highly isolated thickets during years when its predators, the Siberian chipmunk ( *Tamias sibiricus* ), are abundant (Forstmeier and Weiss 2004). Birds breeding in mangroves face two primary nesting choices: building nests in trees, as is often reported for herons that are colonial breeding in mangrove trees (Zou et al. 2008; Tan et al. 2013; Cheng et al. 2019; Mancini et al. 2023), or building nests directly on the ground, as seen in some shorebirds (Gopi and Pandav 2007). However, our experimental results indicate that both tree‐ and ground‐nesting birds in Zhanjiang mangrove habitats experience high rates of predation. Various predators inhabit the mangroves, any of which can lead to reproductive failure. For example, greater coucals and rats prey not only on bird eggs, but also on chicks (Narayana et al. 2013). During low tides, the presence of large numbers of various crabs in the mangroves may threaten only the survival of the offspring of breeding birds with ground nests (Kwon et al. 2018), but at high tides, these crabs may climb up trees and prey on the eggs and even chicks of breeding birds in tree nests (Yang et al. 2014; Kwon et al. 2018). In addition, our survey also found many ant nests in the Zhanjiang mangrove habitats. Although the ants do not necessarily prey on the eggs, they do bite the chicks in some cases, or through ant‐mediated predation (Haemig 1999; Avilés 2024), which can also cause breeding failure. Therefore, the birds that breed in the Zhanjiang mangrove habitats may be exposed to various predation risks posed by multiple predators.

In conclusion, our study confirms that there are multiple nest predators of birds in Zhanjiang mangrove habitats, and birds breeding in Zhanjiang mangrove habitats are exposed to a relatively high risk of predation. Whether higher predation risk leads to fewer birds breeding in the study area remains to be further investigated.

## Author Contributions

**Yiming Liu:** data curation (equal), investigation (lead), methodology (equal), writing – original draft (equal). **Jianping Liu:** formal analysis (lead), validation (equal), writing – review and editing (equal). **Wei Liang:** conceptualization (lead), funding acquisition (lead), supervision (lead), validation (equal), writing – review and editing (equal).

## Funding

W.L. was supported by the specific research fund of The Innovation Platform for Academicians of Hainan Province (no. YSPTZX202408). J.L. was supported by the 2023 Ningxia Hui Autonomous Region Youth Science and Technology Support Talent Training Project.

## Ethics Statement

The experiments comply with the current laws of China. No special permit was required for this study as it was not involved in animal or plant collection.

## Conflicts of Interest

The authors declare no conflicts of interest.

## Supporting information


**Table S1:** ece372651‐sup‐0001‐TableS1.xls.

## Data Availability

Data used for this study is provided as Table S1 and can be found at https://figshare.com/s/65878800ac7012ce363f (doi: 10.6084/m9.figshare.28329860).
